# Synthetic Surface for Expansion of Human Mesenchymal Stem Cells in Xeno-Free, Chemically Defined Culture Conditions

**DOI:** 10.1371/journal.pone.0070263

**Published:** 2013-08-05

**Authors:** Paula J. Dolley-Sonneville, Lori E. Romeo, Zara K. Melkoumian

**Affiliations:** Corning Life Sciences Development, Corning Incorporated, Corning, New York, United States of America; University of California, San Diego, United States of America

## Abstract

Human mesenchymal stem cells (hMSCs) possess three properties of great interest for the development of cell therapies and tissue engineering: multilineage differentiation, immunomodulation, and production of trophic factors. Efficient *ex vivo* expansion of hMSCs is a challenging requirement for large scale production of clinical grade cells. Low-cost, robust, scalable culture methods using chemically defined materials need to be developed to address this need. This study describes the use of a xeno-free synthetic peptide acrylate surface, the Corning® Synthemax® Surface, for culture of hMSCs in serum-free, defined medium. Cell performance on the Corning Synthemax Surface was compared to cells cultured on biological extracellular matrix (ECM) coatings in xeno-free defined medium and in traditional conditions on tissue culture treated (TCT) plastic in fetal bovine serum (FBS) supplemented medium. Our results show successful maintenance of hMSCs on Corning Synthemax Surface for eight passages, with cell expansion rate comparable to cells cultured on ECM and significantly higher than for cells in TCT/FBS condition. Importantly, on the Corning Synthemax Surface, cells maintained elongated, spindle-like morphology, typical hMSC marker profile and *in vitro* multilineage differentiation potential. We believe the Corning Synthemax Surface, in combination with defined media, provides a complete synthetic, xeno-free, cell culture system for scalable production of hMSCs.

## Introduction

Stem cells are one of the most unique cell types in the human body because of their ability to self-renew and differentiate into multiple lineages. Stem cells have been identified in embryonic as well as adult tissues [1]. The groundbreaking work by Freidenstein and colleagues led to the identification of the adherent fibroblast cell population that formed clonal colonies, or colony-forming unit fibroblasts (CFU), when seeded at low density on plastic culture dishes [2]–[5]. The term “mesenchymal stem cells” (MSC) appeared for these cells in the early 1980s [6], although some investigators suggested the term “multipotent mesenchymal stromal cells” [7] instead. MSCs have also been isolated from other tissue sources including adipose [8]–[9], amniotic [10], placenta [10], umbilical cord [11], tendon [12], synovium [13], dental pulp [14], muscle [15]–[16], and skin [17]. MSCs have been shown to differentiate into multiple lineages−adipogenic, osteogenic, and chondrogenic [18]–[19], as well as non-mesenchymal lineages, such as neural [20] and hepatic [21]. Phenotypically, MSCs highly express specific cell surface antigens, such as CD73, CD90, CD105 and lack expression of surface antigens specific for hematopoietic cells, such as CD11b, CD14, CD19, CD34, CD45, and CD79a [22]. MSCs are also capable of *in vivo* and *in vitro* immunomodulation and paracrine effects mediated by their production of a wide range of growth factors and cytokines [23]–[24].

The availability of multiple tissue sources for MSCs, their immunomodulatory and trophic effects, as well as their multi-lineage differentiation capability, have expanded their applications in both cell therapy and tissue regeneration [25]–[31]. Bone marrow is the most documented source for human MSCs. However, the bone marrow aspiration procedure is invasive and hBM-MSCs only represent a small fraction of the total cells isolated from bone marrow aspirates. Fat, amniotic tissue, umbilical cord Wharton’s jelly, and placenta have been shown to provide an alternative source for hMSCs. Fat aspirates obtained from liposuction procedures are becoming an attractive source of hMSCs due to the easy access and high number of hMSCs. About 100 times more MSCs can be isolated from an adipose tissue than bone marrow [32]. Adipose-derived human MSCs (hAD-MSCs) have a greater proliferation potential [33], similar phenotype [34]–[35], differentiation [36], and immunomodulatory potential [32], [37].

Over 142 clinical studies using hMSCs were listed on htpp://www.clinicaltrials.gov website for treatment of multiple conditions, including graft vs. host disease, Chrohn’s disease, severe chronic obstructed pulmonary disease, acute myocardial infarction, type I diabetes, multiple sclerosis, bone loss, cardiac ischemia, osteonecrosis, systemic sclerosis, and liver cirrhosis [38]. The uses of autologous versus allogeneic hMSCs, as well as the dosage and treatment regimens are still being explored. Currently a single dosage for treatment can require 0.4–10x10^6^ cells per kilogram of body weight per patient [39]–[40]. In some cases a patient may need multiple doses throughout a treatment [39], [28]. Tissue sources contain limited number of hMSCs. For example, a typical bone marrow aspirate contains only 0.001–0.01% mononuclear cells of which a smaller fraction is MSCs [19], [41].

Therefore, large-scale production of clinical grade hMSCs is necessary for therapeutic applications. Dos Santos *et al* demonstrated a successful expansion of hMSCs in a scalable microcarrier-based stirred culture system under xeno-free conditions [42].Culture platforms like this will be needed to meet the therapeutic lot size associated with the manufacturing of the adherent cells. Rowley J, *et al* evaluated new platforms available for expanding adherent cells and their potential for meeting lot size requirements, new production methods, and addressing downstream processing bottlenecks [43].

To standardize the isolation, culture and characterization methods for hMSCs, the Mesenchymal and Tissue Stem Cell Committee of the International Society for Cellular Therapy (ISCT) proposed the minimal criteria to define MSCs [22]. The criteria include adherence to plastic; expression of CD105, CD73, CD90; lack of expression of CD45, CD34, CD14, or CD11b, CD79α, CD19, and HLA-DR surface molecules; differentiate into adipocytes, osteoblasts, and chondrocytes in vitro [22]. Traditionally, isolation and culture of hMSCs has been performed on tissue culture treated (TCT) polystyrene in FBS containing medium. Cells cultured in this system show limited expansion of about 15–25 population doublings before they undergo replicative senescence [44]. Furthermore, animal-derived components of this culture system require expensive quality testing to ensure their freedom from pathogens. Other limitations/risks include the lack of reproducibility, and potential source of antigens, which can cause immunogenic reactions in patients. Hence, there is a strong need for development of low cost, robust, scalable cell culture methods for hMSCs using xeno-free, defined materials.

Several xeno-free defined culture systems are commercially available. These culture systems consist of serum-free chemically defined medium supplemented with growth factors, to support cell growth, and biological extracellular matrix (ECM) substrate, to support cell adhesion. The biological materials are expensive to manufacture, have limited scalability and high batch-to-batch variability. Synthetic acrylates are biomaterials with tunable properties that can be modified with biologically active molecules to provide synthetic, scalable alternatives to ECM proteins [45]. The Corning® Synthemax® Surface used in this study is a synthetic surface comprised of an acrylate polymer functionalized with a short peptide sequence derived from the vitronectin protein (PQVTRGDVFTMP), to mimic biological ligands for cell adhesion [45]. Our previous studies demonstrated successful expansion and differentiation of human embryonic stem cells (hESCs) [45] and human induced pluripotent stem cells (hiPSCs) [46] on Corning Synthemax Surface in xeno-free defined media.

In this study we evaluated the ability of the Corning Synthemax Surface to support long-term expansion of hMSCs in serum-free, defined medium. Cell performance on the Corning Synthemax Surface was compared to cells cultured on: a) biological ECM coating in xeno-free defined medium (ECM/serum-free) and b) traditional culture conditions on TCT plastic in FBS-containing medium (TCT/with serum).

## Materials

### Cell Culture

Human bone marrow-derived mesenchymal stem cells (hBM-MSCs) were obtained from STEMCELL Technologies. Human adipose-derived mesenchymal stem cells (hAD-MSCs) were obtained from Lonza Group Ltd. Both hBM-MSCs and hAD-MSCs were expanded in serum-free, xeno-free (XF) conditions (MesenCult®-XF medium and MesenCult®-ACF-XF Attachment Substrate, STEMCELL Technologies) according to the manufacturer’s instructions and used at passage 3 (BM-MSCs) or passage 1 (AD-MSCs) for this study.

MesenCult®-XF, xeno-free, defined Medium (MC-XF) (STEMCELL Technologies), MesenCult®-ACF-XF Attachment Substrate (MC-ASB) (STEMCELL Technologies); STEMPRO® MSC SF Xeno-free medium (Invitrogen), CELLStart™ (Invitrogen), TheraPEAK™ MSCGM-CD (Lonza Group Ltd), Corning® stemgro® hMSC Medium (Corning), Corning® Synthemax® Surface®-T 6 well plate (Corning); Tissue Culture Treated (TCT) Polystyrene 6 well plate (Corning); 200mM L-Glutamine Solution (STEMCELL Technologies), MesenCult®-Dissociation Kit (STEMCELL Technologies), Iscove’s Modified Dulbecco’s Medium (IMDM) (Invitrogen); Fetal Bovine Serum (Invitrogen), 0.05% Trypsin-EDTA Solution (Invitrogen), 1X Dulbecco’s Phosphate Buffered Saline without Ca+^2^ and Mg+^2^ (DPBS) (Invitrogen).

### Flow Cytometry

Staining Buffer with BSA (SB) (BD Pharmigen); Heat Inactivated Fetal Bovine Serum (HI-FBS) (Invitrogen); 4% Paraformaldehyde Solution (PFA)(USB Corp), Antibodies: CD44-HCAM, CD166, CD19, and CD14 (Millipore Chemicon), all corresponding primary isotype controls and isotype specific secondary Alex Flour 488 antibodies (Invitrogen/Molecular Probes).

### *In vitro* Multilineage Differentiation Assays

Poetics™ Adipogenic Differentiation BulletKit® (Lonza Group Ltd); Oil Red Staining Kit (Millipore), Osteogenic Stimulatory Kit (STEMCELL Technologies), Alizarin Red Stain (Millipore), STEMPRO® Chondrogenesis Differentiation Kit (Invitrogen); 1% Alcian Blue Stain (Fisher Scientific).

## Methods

### Attachment and Short Term Growth of HBM-MSCs

Cryogenically preserved p1 human bone marrow-derived MSCs from a single hBM-MSC donor (purchased from SCT)were thawed and seeded according to the manufacturer’s recommended protocol using ECM/xeno-free defined culture condition (MesenCult®-ACF-XF Attachment Substrate/MesenCult®-XF medium). Upon reaching 80% confluence, the cells were subcultured (passage 2) into the following experimental conditions:1) ECM/xeno-free defined culture condition (MesenCult®-ACF-XF Attachment Substrate/MesenCult®-XF medium), 2) Synthemax/xeno-free defined culture condition(Corning® Synthemax® Surface/MesenCult®-XF medium), 3) TCT/xeno-free defined medium (TCT/MesenCult®-XF medium), 4) TCT/with serum (TCT/10% FBS containing medium). The same surface/media designations will be used throughout this study, unless specified otherwise. Two duplicate 6-well plates were used per conditions throughout the study with all 6 wells harvested, pooled, and counted three times. The cultures were re-fed on day 3. Cells were harvested 24 and 96 hours post-seeding with 0.05% Trypsin-EDTA to assess cell plating efficiency (% of attached cells after 24 hours) and cell proliferation at 80−90% monolayer confluence, respectively. Viable cell number was assessed using Trypan Blue dye exclusion assay with the ViCell XR Cell Viability Analyzer (Beckman Coulter Inc.); 3 counts per each pooled sample were performed and then averaged. To determine statistical significance, an ANOVA followed by Tukey’s method, was performed on the viable cells per cm^2^ against the various surface conditions. We consider a p-value less than 0.05 to be statistically significant.

### Long-Term Multi-Passage Growth of HBM-MSCs

Prior to the initiation of the multi-passage study we evaluated several plating densities based on recommendation of the manufacturer of hBM-MSCs used in the study (AllCells) and MesenCult xeno-free defined medium (SCT). The recommended range was 3000–10,000 cells cells/cm^2^. Specifically, we tested plating densities in a range of 2500–10,000 cells/cm^2.^ Based on the literature, higher seeding densities are recommended for xeno-free defined media compared to serum-containing media [47]–[49]. The plating density of 7000 cells/cm^2^ gave the best cell fold expansion and was chosen for the hBM-MSC short-term and long-term studies.

Cryogenically preserved p1 human bone marrow-derived MSCs from a single hBM-MSC donor (purchased from SCT) were thawed and seeded according to the manufacturer’s recommended protocol using ECM/xeno-free defined culture condition (MesenCult®-ACF-XF Attachment Substrate/MesenCult®-XF medium). Upon reaching 80% confluence, the cells were subcultured (passage 2) into the following experimental conditions:1) ECM/xeno-free defined culture condition (MesenCult®-ACF-XF Attachment Substrate/MesenCult®-XF medium), 2) Synthemax/xeno-free defined (Corning® Synthemax® Surface/MesenCult®-XF medium), 3) TCT/with serum (TCT/10% FBS containing medium). After a brief adaptation phase of 5 days, the cells were sub-cultured (passage 3) into the same conditions for the multi-passage study. Two duplicate 6-well plates were used per conditions throughout the study with all 6 wells harvested, pooled, and counted three times. Cells were sub-cultured, using MesenCult Dissociation/Inhibitor Solutions for the xeno-free defined serum-free conditions and 0.05% Trypsin-EDTA for the TCT/FBS condition, around 80−90% monolayer confluence (days 4−6) for 8 serial passages. A full media exchange was performed on day 3 only if additional time in culture was needed to meet the desired confluence for harvest and passaging. Seeding density of 7000 cells/cm^2^ and 6 well plate vessel format was used throughout the study. Cell morphology, viability, cell number, population doublings, and doubling time were assessed at each passage for all experimental conditions, based on average of 3 counts per each pooled wells for each condition. Cumulative net cell number was calculated by multiplying the initial seeding cell number of 70,000 cells/well at passage 1 by the fold expansion at each passage for 8 serial passages. The following formulas were used to calculate the doubling time (DT) and population doublings (PDs), respectively:

DT (hours) = (days in culture × 24 hours/day) × LN(2)/LN(N/X).

N = Total number of cells harvested.

X = Initial number of cells seeded.

PD = 3.321(logN-logX).

N = Total # of cells harvested.

X = Initial number of cells seeded.

To determine statistical significance, an ANOVA followed by the Tukey’s method was performed on the doubling time (7 passages) and % viability (8 passages). This study was performed in two independent experiments using cells from two different donors.

For each experimental condition cells were fixed in 2% PFA in DPBS (1x10^6^ cells/ml of 2% PFA) for 10 minutes at room temperature. Cells were incubated in blocking buffer (10% heat-inactivated FBS in staining buffer) for 15 min at 4 °C, followed by 30 minute incubation at 4°C with primary antibodies (10 µg/ml antibody per 1x10^6^ cells) against CD44, CD166, CD19, CD14 or corresponding isotype controls (MsIgG, MsIgG1, MsIgG2a) in blocking buffer. After a brief wash, cells were incubated with 1∶800 dilution of corresponding secondary antibodies (goat anti-mouse IgG, IgG1, or IgG2a Alexa Fluor 488) in staining buffer for 30 minutes at 4°C in the dark. For each sample, 30,000 events were acquired using BD FACS Calibur Flow Cytometer (BD Biosciences). Histogram overlay subtraction analysis using the BD Cell Quest Pro software (BD Biosciences) was performed to calculate the percent of marker positive cells.

HBM-MSCs were differentiated into adipocytes using the Adipogenic Differentiation Kit (Lonza’s Poetics™) according to the manufacturer’s protocols. Briefly, after 9 passages on Corning® Synthemax® Surface in xeno-free defined medium, hBM-MSCs were seeded at the density of 21,000 cell/cm^2^ in 6 well plates and allowed to reach 100% confluence with media exchange every 2−3 days. The cells were then induced in three sequential induction/maintenance cycles according to the protocol. At the end of the differentiation (20 days post induction), cells were fixed with 2% PFA for 10 minutes at room temperature and then stained with Oil Red O stain. The extent of adipogenic differentiation was determined microscopically by the appearance of red stained lipid vacuoles.

HBM-MSCs were differentiated into osteocytes using the Osteogenic Stimulatory Kit (STEMCELL Technologies) according to the manufacturer’s protocols. Briefly, after 9 passages on Corning® Synthemax® Surface in xeno-free defined medium, hBM-MSCs were seeded at the density of 7,000 cell/cm^2^ in 6 well plates and allowed to reach 70−80% confluence with media exchange every 2−3 days. The cells were then induced by adding the osteogenic medium, which was exchanged every three days over a period of 21 days. The cells were fixed with 3% PFA at room temperature for 30 minutes and stained with Alizarin Red to visualize the presence of calcium deposits in the mineralized material produced by bone cells. Extent of the differentiation was determined microscopically by the appearance of red stained calcium phosphates.

HBM-MSCs were differentiated into chondrocytes using the STEMPRO® Chondrogenesis Differentiation Kit (Invitrogen) according to the manufacturer’s protocols. Briefly, after 9 passages on Corning® Synthemax® Surface in xeno-free defined medium, hBM-MSCs were diluted in the same medium to a concentration of 1.6x10^7^cells/cm^2^ and applied as droplets to 6 well plates with the Corning Synthemax Surface. The plates were incubated at room temperature under sterile conditions at 100% humidity for two hours. The culture medium was then replaced with warm chondrogenic differentiation medium, which was exchanged every two days over a period of 14−16 days. The cells were then fixed with 2% PFA for 30 minutes at room temperature and stained with Alcian Blue for the presence of proteoglycans synthesized by chondrocytes. The extent of the differentiation was determined microscopically by the appearance of blue stained proteoglycans.

### HBM-MSCs Culture on the Corning Synthemax Surface in Several Commercially Available Defined Media

Cryogenically preserved p1 human bone marrow-derived MSCs from a single donor (SCT) were thawed and seeded directly into four different commercial xeno-free serum-free defined media using a culture surface recommended by the media manufacturer: 1) MesenCult®-XF Complete Medium (SFXF) and MesenCult®-XF Attachment Substrate (MC-ASB) coated T75 flasks; 2) STEMPRO® MSC SF Xeno-free medium and CELLStart™ coated T75 flasks; 3) TheraPEAK™ MSCGM-CD and TCT T75 flasks; 4) Corning® stemgro® hMSC Medium and Corning® CellBIND® Surface. After a brief adaptation phase for 2 passages, the cells were seeded (passage 3) at the density of 3500 cells/cm^2^ in duplicate 6 well plates per condition, using either control surface (recommended by the media manufacturer) or Corning® Synthemax® Surface for each commercial media listed above. Cells were refed on day 3 and harvested at ∼80−90% confluence. Viable cell number, % viability, population doublings, and doubling time was assessed using Trypan Blue dye exclusion assay with the ViCell XR Cell Viability Analyzer (Beckman Coulter), as described in previous methods’ sections.

### HAD-MSC Growth on the Corning Synthemax Surface

Similar plating density optimization experiment was performed as described for hBM-MSCs. The plating density of 3500 cells/cm^2^ gave the best cell fold expansion and was chosen for hAD-MSC long-term growth studies.

Frozen hAD-MSCs at passage 1 were thawed into the same experimental conditions (single culture vessel per each condition), as described for hBM-MSCs above: 1) ECM/xeno-free defined, 2) Synthemax/xeno-free defined 3)TCT/with serum. After a brief adaptation of five days, the cells were sub-cultured for three sequential passages using the same three culture conditions, 1 culture vessel per each condition. A seeding density of 5000 cells/cm^2^ and 75 cm^2^ flask format per condition were used for this study. The viable cell number was determined at each passage using Trypan Blue dye exclusion assay with the ViCell XR Cell Viability Analyzer; 3 counts per vessel and averaged.

## Results

### Attachment and Short Term Growth of HBM-MSCs on the Corning® Synthemax® Surface

**Figure 1:**
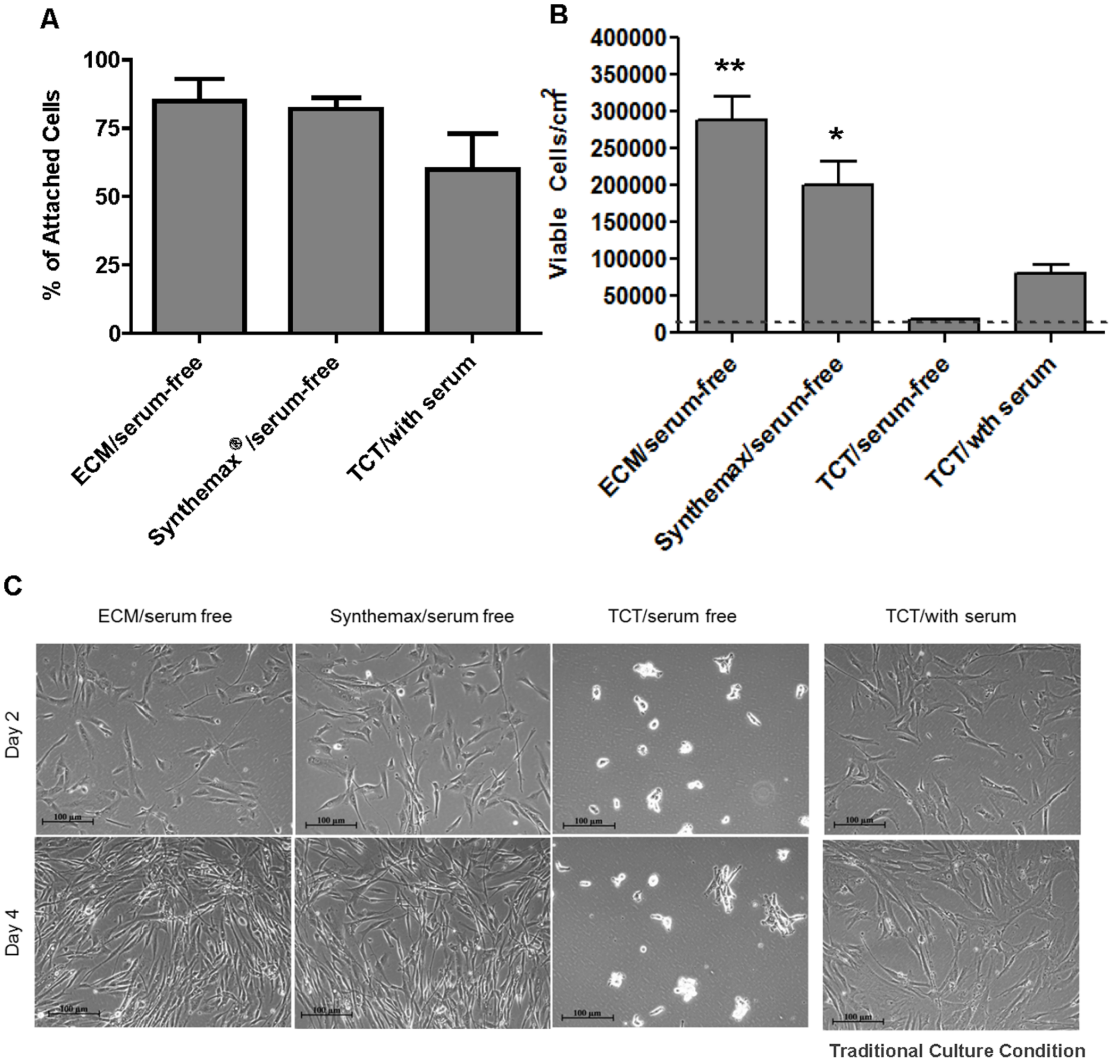
Attachment and short term growth of hBM-MSCs in different surface/media conditions. 7000 cells/cm^2^ were seeded in 6 well plates using the indicated conditions. Cells were harvested after 24 (A) and 96 (B) hours to determine the plating efficiency and cell expansion, respectively. The dotted line in (B) indicates the seeding density. The error bars in A and B represent the average +/− STDEV from 2 (panel A) and 5 (panel B) independent experiments. Statistical analyses by an ANOVA followed with a Tukey’s method performed on the viable cells per cm^2^ (B) found that the samples are statistically different from each other (p-value = 0.000) and that the ECM (**) is higher than both the Synthemax and TCT/serum, and Synthemax (*) is higher than TCT/serum condition. (C) The representative cell morphology micrographs for the indicated surface/media conditions. Scale bar is 100 µm.

Attachment of hBM-MSCs to tissue culture plastic is facilitated by the adsorption of ECM proteins present in the serum to the plastic surface. Under serum-free culture conditions the coating of culture surfaces with ECM proteins enables hBM-MSC attachment and spreading. Therefore, we tested if Synthemax Surface could replace ECM proteins to support the attachment of hBM-MSCs in xeno-free defined medium. Attachment efficiency, calculated as percent of attached cells 24 hours after seeding, was examined for cells seeded on the Corning Synthemax Surface, ECM-coated surfaces and TCT. As shown in Figure 1A, the Corning® Synthemax® Surface supports efficient attachment of cells in xeno-free defined medium. Attachment efficiency on Corning Synthemax Surface (82%) was comparable to that on ECM-coated surface (85%) and higher than on TCT surface (60%) in serum-containing medium. In the absence of serum, hBM-MSCs did not attach to TCT surface, confirming the requirement of adhesion-promoting factors for attachment of these cells.

Next, we wanted to examine the proliferation of hBM-MSCs on the Corning Synthemax Surface in xeno-free defined medium. As shown in Figure 1B, after four days in culture on the Corning Synthemax Surface, cells expanded from 7,000 cells/cm^2^ (seeding density) to 200,000 cells/cm^2^ (harvesting density). The fold expansion on the Corning Synthemax Surface (29 fold) was significantly higher than for traditional hBM-MSC culture conditions on TCT in serum-containing medium (12 fold). An ANOVA performed on the viable cells per cm^2^ (Figure 1, Panel B) and it was found that the samples are statistically different from each other (p = 0.000). In this case, Tukey’s method was performed and it was found that the ECM results are higher than both the Synthemax and the TCT, and the Synthemax is higher than the TCT. This is probably due to the higher concentration of growth factors present in the xeno-free defined medium, since hBM-MSC expansion on the Corning Synthemax Surface in serum-containing medium was comparable to TCT (data not shown). Slightly lower expansion of cells on the Corning Synthemax Surface compared to ECM in xeno-free defined medium is probably due to the adaptation response during the transition of cells from ECM to Corning Synthemax Surface (prior to the experiment, the cells were maintained on ECM surface). TCT surface in xeno-free defined medium did not support expansion of hBM-MSCs.

The representative hBM-MSC morphology for the described experimental conditions is shown in Figure 1C. Cells cultured on TCT plastic in serum medium exhibited typical elongated, fibroblast-like hMSC morphology. In the absence of serum on TCT plastic surface, a small percentage of cells formed loosely attached 2–5-cell clusters, with little to no spreading. Cells on the Corning® Synthemax® Surface and ECM-coated surface had more elongated morphology, which allowed achievement of higher cell confluence compared to cells cultured on TCT in serum. In summary, our results demonstrate that the Corning Synthemax Surface supports efficient attachment and short term growth of hBM-MSCs in serum free, xeno-free chemically defined medium.

### Long-Term Multi-Passage Growth of HBM-MSCs on the Corning Synthemax Surface in Serum-free Defined Medium

**Figure 2:**
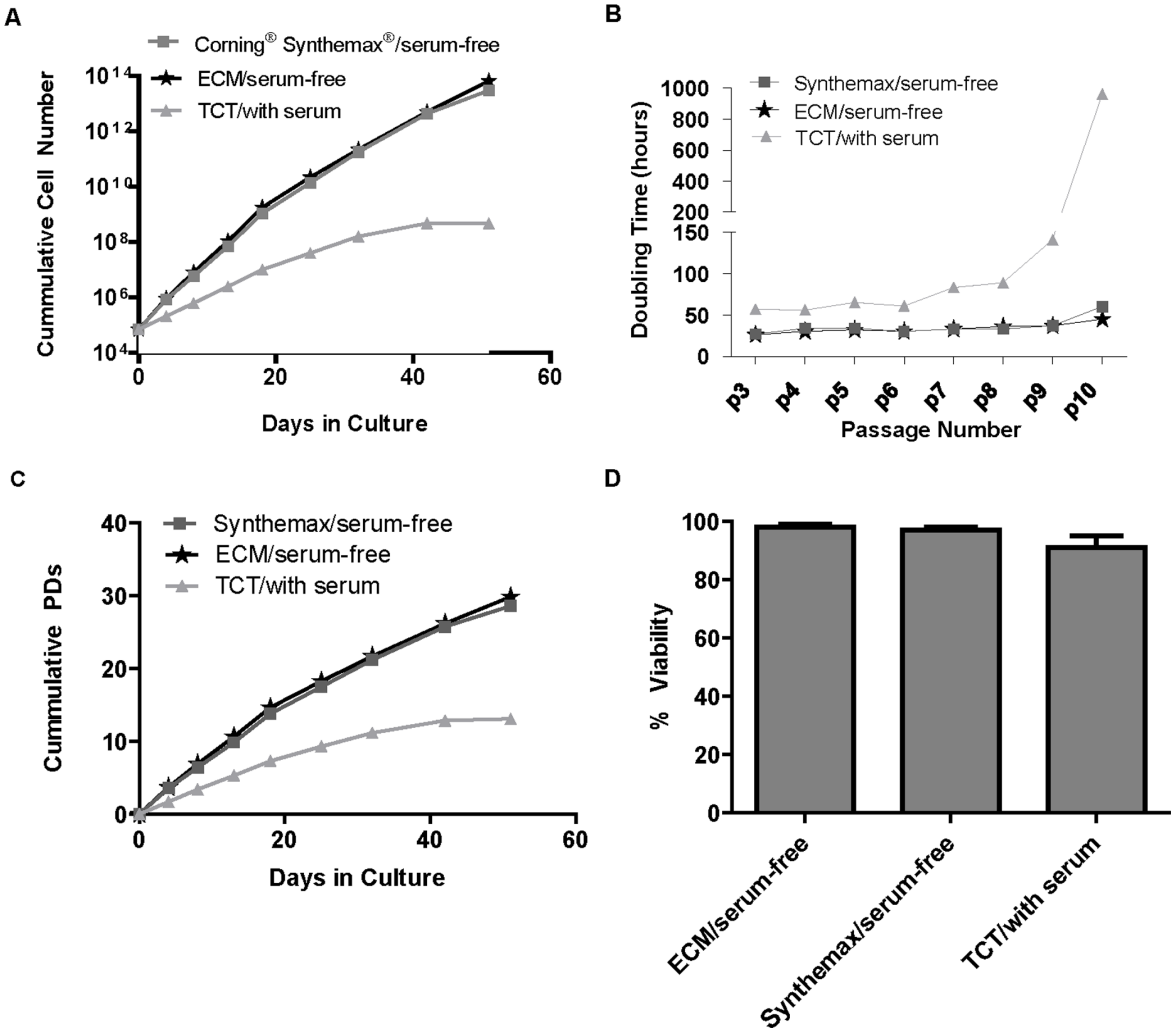
Long-term multipassage culture of hBM-MSCs in different surface/media conditions. Cells were cultured for 8 sequential passages using the indicated conditions. Seeding density at each passage was 7000 cells/cm^2^. Cumulative cell number (A), doubling time (B), cumulative population doublings (PDs) (C), and % viability (D) across the multiple passages are shown. The error bars in D represent the average +/− STDEV from 7 serial passages. Statistical analyses by an ANOVA followed by the Tukey’s method performed on the doubling time (7 passages) and % viability (8 passages) found that doubling time of TCT/with serum was significantly higher and that the % viability for TCT/with serum was significantly lower compared to the ECM and Synthemax Surface serum-free conditions (p-value = 0.000).

Next, we examined if the Corning Synthemax Surface can support long-term culture of hBM-MSCs in xeno-free defined medium. Cells were maintained for 8 sequential passages (about 50 days) using the following experimental conditions: 1) Corning Synthemax Surface in xeno-free defined medium, 2) ECM coated surface in xeno-free defined medium, 3) TCT surface in serum-containing medium (traditional condition). Projected cumulative cell number was calculated across multiple passages. As shown in Figure 2A, significantly higher projected cell number (100-fold higher after 20 days and 10,000 higher after 45 days) was observed on the Corning Synthemax Surface and ECM in xeno-free defined medium compared to TCT/serum condition. Consistent with the higher cell number, cells on the Corning Synthemax Surface and ECM in xeno-free defined medium had shorter doubling times (averaging 35 and 33 hours, respectively) compared to cells in TCT/serum (ranged from 57 to 960 hours during 8 passages, Figure 2B). An ANOVA of doubling time, Figure 2 Panel B, was performed on 7 passages, and it was determined that the samples were statistically different from each other (p-value = 0.000). Tukey’s method was performed and it was found that the TCT/serum condition had statistically higher doubling time than the remaining two conditions. After 40 days in culture (passage 10), cells for TCT/serum condition reached replicative senescence, while cells on the Corning Synthemax Surface and ECM in xeno-free defined medium continued to proliferate. Importantly, cells maintained high viability (97%) throughout the long-term culture on the Corning Synthemax Surface (Figure 2D). An ANOVA test was performed on % viability over 8 passages (Figure 2 Panel D), and it was determined that the samples were statistically different from each other (p-value = 0.000). Tukey’s method was performed and it was found that the TCT/serum condition had statistically lower % viability than the remaining two conditions. In summary, our results demonstrate successful maintenance of hBM-MSCs on the Corning® Synthemax® Surface in serum-free, xeno-free defined medium. Calculated cell expansion was significantly higher compared to the traditional hBM-MSC culture method on TCT in serum-containing medium.

This study was focused on the expansion of hMSC in xeno-free, defined culture conditions. The isolation from different tissue sources is another important step in manufacturing of clinical grade cells. It would be interesting to see if the same platform using Synthemax Surface and xeno-free, defined medium will support hMSC isolation.

### HBM-MSCs Retain Surface Antigens after Long Term Culture on the Corning Synthemax Surface

**Figure 3:**
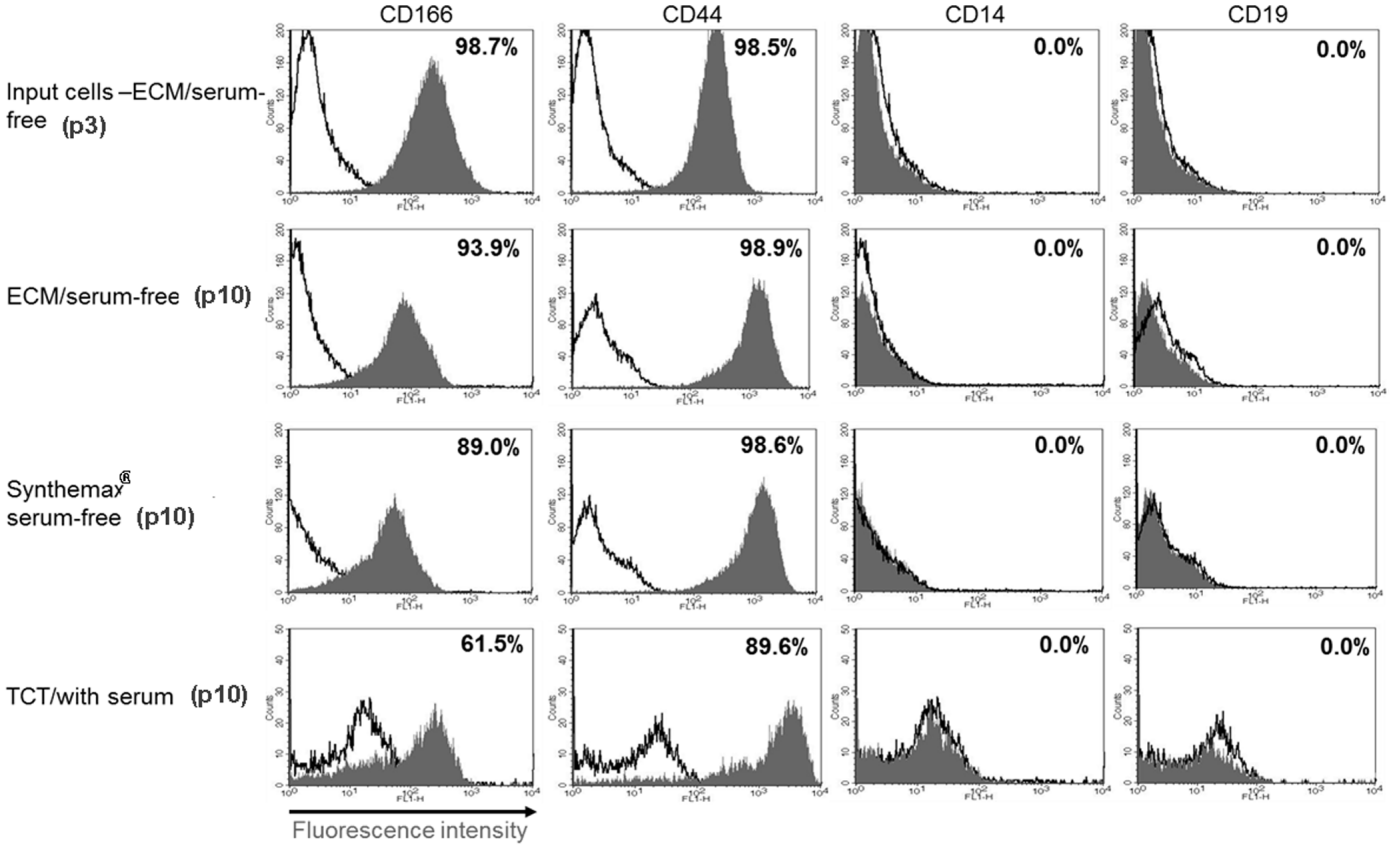
Cell surface antigen expression in hBM-MSC cultured in different surface/media conditions. Flow cytometry histograms for the indicated surface antigens are shown for the input cells (passage 3) and cells cultured using the indicated surface/media conditions for 7 passages.

Surface antigen expression is one of the important criteria for characterization of hBM-MSC identity [50]. The Mesenchymal and Tissue Stem Cell Committee of the International Society for Cellular Therapy (ISCT) proposed the following surface antigens for hMSCs: expression of CD105, CD73, CD90, and lack of expression of CD45, CD34, CD14, or CD11b, CD79α, CD19 and HLA-DR [45]. Since the establishment of the ISCT criteria, other cell surface antigens, such as CD166 were described for hMSCs [51]–[54]. The expression levels of these antigens can vary during the culture of hBM-MSC and need to be carefully monitored upon introduction of any changes in hBM-MSC culture protocols. Therefore, we examined the expression of two highly expressed (CD166 and CD44) and two not-expressed (CD 14 and CD19) surface antigens for hMSC characterization after long term culture of cells on the Corning Synthemax Surface. Figure 3 shows flow cytometry histogram overlays for the described surface antigens across all three experimental conditions. Input cells, designated as passage 3, represent the initial hBM-MSC population used for this study. As shown in Figure 3, cells grown on the Corning Synthemax Surface in xeno-free defined medium retained high levels of CD166 and CD44 and no expression of CD14 and CD19 surface antigens after 7 consecutive passages. Similar results were obtained for cells cultured on ECM-coated surface in xeno-free defined medium. In contrast, cells cultured in serum-containing medium for 8 passages showed decrease in differentiation potential (data not shown), which correlated with decrease in the expression of CD166 and CD44 markers, increase in cell doubling time, and senescent morphology. Although at this point we do not understand why the expression of these two markers decrease in cells cultured in serum-containing medium, it could be simply a result of cellular senescence observed after prolonged culture of MSCs under this condition. The second multi-passage study was performed on Synthemax Surface with different xeno-free, defined medium (Corning ® stemgro® hMSC medium) and different hBM-MSC donor. The similar results were observed as with the MesenCult medium (data not shown). In this study we analyzed additional MSC surface markers: CD73, 90, 105. All three markers were expressed at >95% after multiple passages in Synthemax/stemgro condition (data not shown). Furthermore, similar results were obtained for hMSCs cultured on Synthemax-coated microcarriers in xeno-free, defined media using hMSCs from two different donors (the manuscript is in preparation).

Our results demonstrated that after long term culture on the Corning Synthemax Surface in xeno-free defined medium, hBM-MSCs still retain the typical surface antigens expression profile.

### HBM-MSCs Retain Multipotency after Long Term Culture on the Corning Synthemax Surface

**Figure 4:**
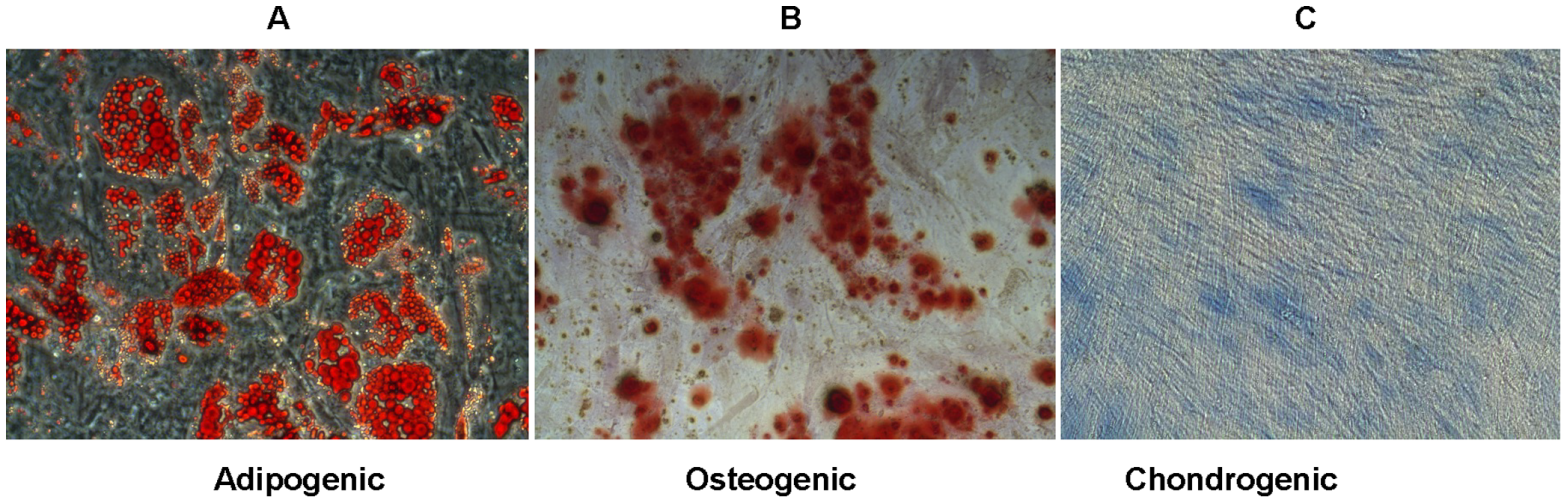
Trilineage differentiation of hBM-MSC after long-term culture on Corning® Synthemax® Surface in xeno-free defined medium. Cells were cultured on Corning Synthemax Surface for 9 passages prior to the induction of adipogenic, osteogenic, and chondrogenic differentiations. (A) Oil Red O staining of lipid droplets after 20 days of adipogenic induction, (B) Alizarin Red staining of calcium phosphate deposits produced by osteocytes after 21 days of osteogenic induction, (C) Alcian Blue staining of proteoglycans synthesized by chondrocytes after 14 days of chondrogenic induction.

Multipotency, or trilineage mesoderm differentiation potential, is another important characteristic of hBM-MSC identity. To examine the multipotency status of hBM-MSCs after long term culture on the Corning Synthemax Surface in xeno-free defined medium, cells at passage 9 were differentiated to adipocytes, chondrocytes and osteocytes on the Corning Synthemax Surface using commercially available differentiation kits. Figure 4 Panel A image shows accumulation of cytoplasmic lipid droplets, visualized by Oil Red O staining and indicative of adipogenic differentiation. Figure 4 Panel B image shows Alizarin Red staining of mineralized deposits secreted by osteocytes differentiated from hBM-MSCs. Finally, Figure 4 Panel C shows Alcian Blue staining of proteoglycans produced by HBM-MSC derived chondrocytes. In contrast, cells cultured in serum-containing medium for 8 passages showed decrease in differentiation potential (data not shown) which correlated with decreased expression of CD166 and CD44 markers, increase in cell doubling time and senescent morphology. Our results showed that hBM-MSCs cultured on the Corning® Synthemax® Surface for 9 passages (>50 days) in xeno-free defined medium, retained the ability to differentiate to adipocyte, chondrocytes and osteocytes.

### Growth of HBM-MSCs on Synthetic Surface in Various Commercially Available Chemically Defined Media

**Figure 5:**
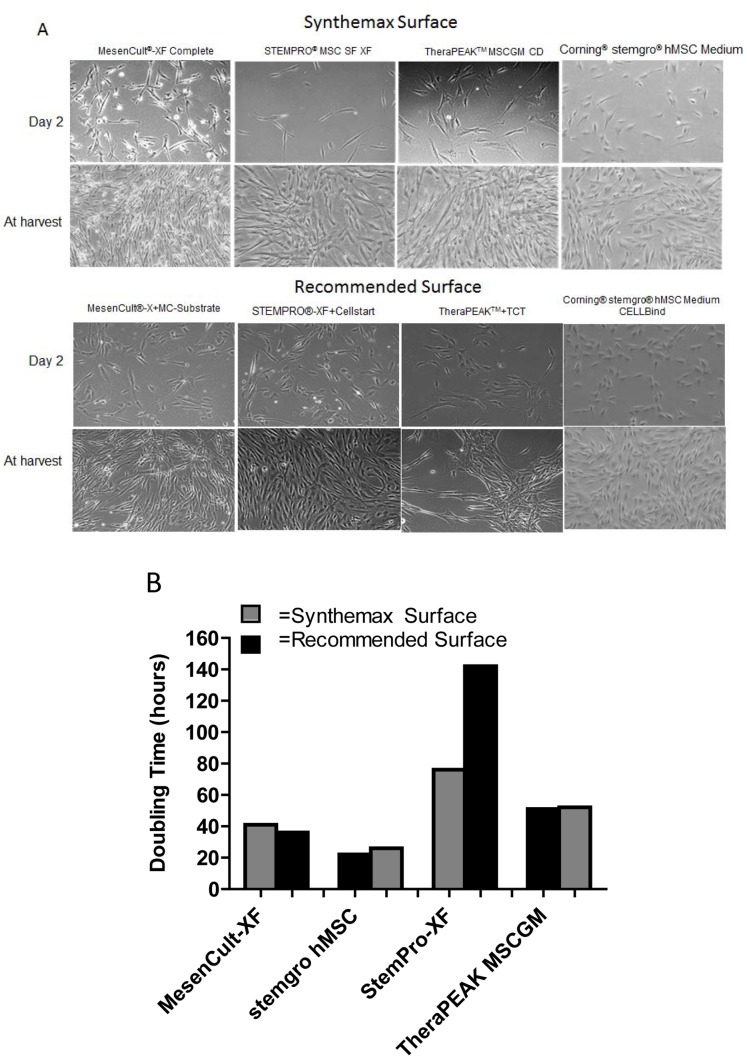
hBM-MSC culture on Corning® Synthemax® Surface in different commercially available serum-free, defined media. Cells were seeded at the density of 3500 cells/cm^2^ in 6 well plates with Corning Synthemax Surface in the indicated media and allowed to expand to 80–90% confluence prior to harvest. (A) Phase contrast micrographs show cell morphology at 48 hours and prior to harvest for different media. (B) Doubling time for cells cultured on Corning Synthemax Surface in different media condition for one passage (3–7 days depending on the media).

The results described above demonstrated successful culture of hBM-MSCs on the Corning Synthemax Surface in a single serum-free, xeno-free defined medium. We wanted to expand this study to additional xeno-free defined media available from different commercial sources. For this experiment, hBM-MSCs were first pre-conditioned to each new medium for one passage using the culture surfaces recommended for the corresponding medium, as described in the Materials and Methods section. The cells were then seeded onto the Corning Synthemax Surface or the corresponding control surface in four different xeno-free defined media and allowed to reach 80% confluence prior to harvest. As shown in Figure 5A, hBM-MSCs attached, spread and proliferated on the Corning Synthemax Surface in all four serum-free media. We observed that cell morphology, time to reach 80–90% confluence (Figure 5 bottom image) and cell doubling time (Figure 5B) varied among different media. This is likely due to the differences in media composition, because similar cell morphologies were observed on the corresponding control surfaces for each medium (data not shown).

Our results showed that the Corning Synthemax Surface can support attachment and expansion of hBM-MSCs in 4 different commercially available serum-free, xeno-free, defined media for mesenchymal stem cells.

### Corning Synthemax Surface Supports Long Term Culture of hAD-MSC in Serum-free, Xeno-free Defined Medium

Besides bone marrow, hMSCs have been isolated from other tissue sources, including adipose, amniotic, umbilical cord, placenta and others. We examined if the Corning® Synthemax® Surface can support serum-free culture of hMSCs from these additional tissue sources.

**Figure 6:**
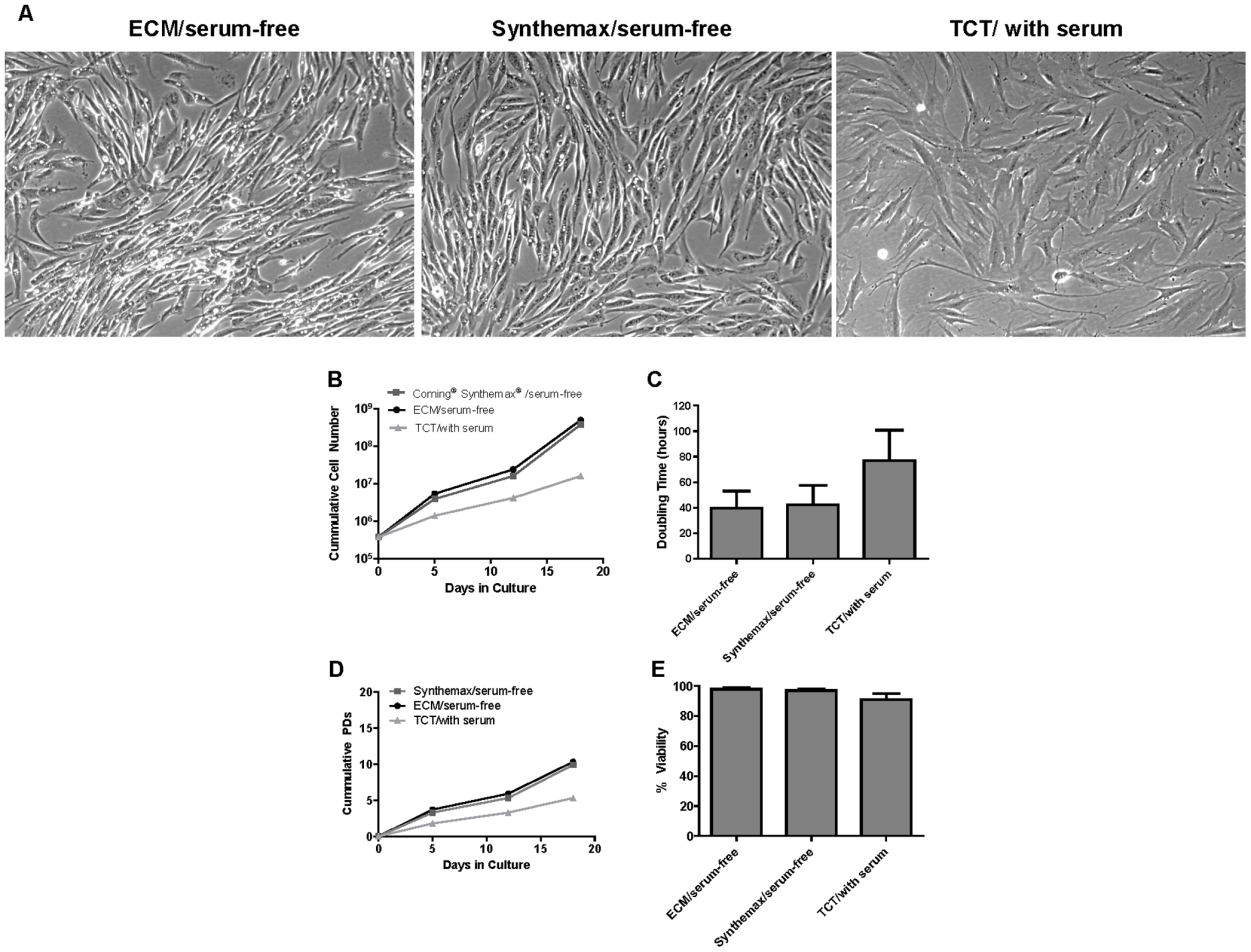
Long-term culture of hAD-MSCs in different surface/media conditions. Cells were cultured for 3 sequential passages in T75 flasks using the indicated conditions. Seeding density at each passage was 5000 cells/cm^2^. Phase contrast micrographs with representative cell morphology on day 6 (A) cumulative cell number (B), doubling time ± STDEV (C), cumulative population doublings (PDs) (D), and % viability ± STDEV (E) across 3 passages, are shown for the indicated conditions.

Human adipose derived stem cells (hAD-MSCs) were cultured on the Corning Synthemax Surface for three serial passages in xeno-free defined medium. Cell expansion was compared to hAD-MSCs cultured on ECM-coated surface in xeno-free defined medium and on TCT in serum-containing medium, similar to our studies with hBM-MSCs. Similar to hBM-MSCs we observed more elongated morphology for hAD-MSCs cultured in xeno-free defined medium compared to serum-containing medium (Figure 6A). Cumulative net cell number for the Corning Synthemax Surface was comparable to that on ECM-coated surface and higher than for cells maintained on TCT in serum-containing medium (Figure 6B). In consistence, with higher cell number, shorter doubling time was observed for cells maintained on the ECM and Corning Synthemax Surface compared to those on TCT in serum containing medium (Figure 6C). Cell viability was >90% for all three experimental conditions (Figure 6E). These results are consistent with our data with hBM-MSCs and show that the Corning Synthemax Surface can support serum-free culture of hMSCs from bone marrow and adipose tissues.

## Discussion

Mesenchymal stem cells may have paved the way for adherent stem cell applications in cell therapy and regenerative medicine, but manufacturing of these cells to support such clinical applications remains challenging by limited *in vitro* expansion of these cells in culture, use of animal derived cell culture materials, and lack of scalability of culture methods. It is clear that further development towards chemically defined, xeno-free culture systems would be beneficial and enabling for successful production and commercialization of hMSCs for therapeutic applications.

In this study we describe the use of the Corning Synthemax Surface for efficient expansion of hMSCs in serum-free defined media. The Corning Synthemax surface is made of synthetic peptide-acrylate material, cGMP manufactured and terminally sterilized by gamma irradiation to provide a sterility assurance level (SAL) of 10^−6^.

HMSCs attached and expanded on Corning® Synthemax® Surface for multiple passages. Projected long term cell expansion on Corning Synthemax Surface was comparable to that on an ECM substrate, and significantly higher (10,000 fold difference) than cells grown in traditional serum-containing medium on TCT plastic. Other studies showed more rapid proliferation of hMSCs in defined media supplemented with growth factors compared to serum-containing medium [55]–[56]. It is well established in the field that hMSCs undergo replicative senescence with decreasing proliferation and changes in cell morphology [35]. The pace of senescence can be affected by cell culture conditions, including seeding density [57], culture medium [44], oxygen concentrations [58], and culture substrate [59]. We found progressive increase in cell doubling time from 61 hours (passage 4) to more than 89 hours (passage 6 and above) when hMSCs were cultured in serum-containing medium. In contrast, cells cultured on the Corning Synthemax Surface in serum-free defined medium showed much shorter doubling time, which remained relatively consistent during the first seven passages and began to increase only at passage 8. The higher proliferation rate and delayed senescence is probably associated with the higher concentrations of growth factors present in the defined medium, while the Corning Synthemax Surface enabled cell adhesion under serum-free environment. Alternatively, some undefined factors in serum may contribute to the shorter life span of hMSCs in serum-supplemented medium. In addition to faster proliferation, we also observed differences in cell morphology when cells were cultured in serum-free versus serum-containing medium. hMSCs grown on the Corning Synthemax Surface and ECM in xeno-free defined medium exhibited more spindle-shaped, elongated morphology relative to the larger more irregularly shaped cells in serum-containing medium. This observation is in line with other studies reporting similar changes in hMSC morphology upon transition to serum-free environments [49].

Changes in hMSC culture conditions, such as culture surface or medium, can affect cell identity. Salasznyk, RM *et al* found that culturing hMSCs on vitronectin and collagen I substrates can promote their osteogenic differentiation [60]. Healy, KE *et al* showed that BSP-RGD functionalized surface is a mild promoter of BM-MSC [50]. The Corning Synthemax Surface is comprised of an RGD-containing 15-mer peptide sequence derived from human vitronectin protein. After 9 passages on Corning® Synthemax® Surface in defined medium, hMSCs were able to differentiate into adipocytes, chondrocytes and osteocytes while retaining their typical MSC immunophenotype (high levels of CD166 and CD44, negative for CD14 and CD19 markers). In contrast, cells cultured in serum-containing medium for 8 passages showed decrease in the expression of CD166 and CD44 markers, which correlated with the increase in cell doubling time, senescent morphology, and decreased differentiation potential (data not shown). Although at this point we do not understand why the expression of these two markers decrease in cells cultured in serum-containing medium, it could be simply a result of cellular senescence observed after prolonged culture of MSCs under this condition. These results indicate that the Corning Synthemax Surface supports efficient expansion of bone marrow derived hMSCs in defined medium while maintaining the important MSC characteristics of trilineage multipotency and phenotype. We expanded this study to three additional hMSC serum-free commercial media and showed that the Corning Synthemax Surface supports hMSC adhesion and expansion in all three media.

Most of the commercial serum-free hMSC media require pre-coating of culture vessels with ECM proteins, to promote cell adhesion in serum-free environment. Different ECM materials, such as fibronectin, gelatin, collagen, and laminin [61] have been shown to support attachment and/or growth of MSCs in xeno-free defined media. However, the use of these materials for scalable production of clinical grade cells is undesirable due to batch to batch variability, potential immunogenicity and labor intensive coating protocols. Many other synthetic materials have been shown to support hMSC adhesion, growth, and functionality, but these materials require serum-containing medium [62]–[67]. In contrast, the Corning Synthemax Surface enables efficient expansion of bone marrow derived hMSCs in serum-free defined media without the need for ECM coating.

We were able to successfully apply Synthemax Surface coating to the larger planar cell culture vessels such as CellSTACK and HYPERFlask (scale-out), as well as microcarriers (scale-up) for large scale production of hMSCs. Our results showed efficient expansion of BM-MSCs on Synthemax coated microcarriers in serum-free, xeno-free, defined media (the manuscript is in preparation).

We believe the combination of the Corning® Synthemax® Surface and xeno-free defined media will provide a complete culture system for efficient expansion of hMSCs for therapeutic applications.
